# A study on the differences in social-face sensitivity and conspicuous consumption tendency based on sports consumers’ impulse buying tendency: focusing on Generation Z

**DOI:** 10.3389/fpsyg.2026.1764875

**Published:** 2026-03-26

**Authors:** Kwon-Hyuk Jeong, Jiung You

**Affiliations:** 1 Department of Taekwondo, College of Physical Education, Kyung Hee University, Yongin-si, Republic of Korea; 2 Department of Sports Science, Hankyong National University, Anseong-si, Republic of Korea

**Keywords:** conspicuous consumption tendency, Generation Z, impulse-buying tendency, MANOVA, social-face sensitivity

## Abstract

**Introduction:**

This cross-sectional study examines whether differences in social-face sensitivity and conspicuous consumption tendencies emerge among South Korean Generation Z sports consumers according to their propensity for impulse buying. While prior research has examined these constructs independently, their integration within digitally mediated sports consumption contexts remains limited. Drawing upon impulse-buying theory and social-face theory, this study investigates whether dispositional impulse-buying propensity is systematically associated with socially evaluative sensitivity and identity-expressive consumption orientations.

**Methods:**

Data were collected from 234 South Korean Gen Z consumers and analyzed using one-way MANOVA.

**Results:**

Results indicated that the high impulse-buying group scored significantly higher in public self-consciousness, social self-shaming tendency, and all sub-factors of conspicuous consumption, whereas no significant difference was found in social formality.

**Discussion:**

These findings suggest statistically significant group-based associations between impulse-buying propensity and both face-related sensitivity and conspicuous consumption orientations within the collectivist cultural context of South Korea. By positioning impulse-buying propensity as a dispositional segmentation variable linked to socially visible consumption patterns, this study contributes to the theoretical integration of impulse-buying and social-face perspectives in sports marketing research. However, given the cross-sectional design, the findings should be interpreted as relational rather than causal.

## 1 Introduction

Generation Z (hereafter Gen Z) refers to those born between 1995 and the early 2010s (Chaturvedi et al., 2020; Novita and Lina, 2024). Generation Z is often described as digital natives, being deeply immersed in digital communication (Axcell and Ellis, 2023). With the rapid advancement of technology, two digitally-centric generations have emerged as the new core consumer base (Zaki, 2019). In particular, consumer research indicates that 41% of Generation Z and 34% of Millennials exhibit a higher propensity for impulse buying than Generation X (32%) and earlier generations (Lina et al., 2022).

Beyond technological immersion, Generation Z consumers are embedded in digitally mediated environments characterized by continuous peer comparison and real-time social feedback. Such environments may be associated with higher levels of public self-consciousness and evaluative sensitivity, as individuals become increasingly aware of how they are perceived by others (Vogel et al., 2014; Nesi and Prinstein, 2015). Research grounded in social comparison theory suggests that exposure to social media feedback mechanisms (e.g., likes, comments, follower metrics) have been linked to heightened concerns regarding social approval and image maintenance (Festinger, 1954; Vogel et al., 2014). Therefore, social-face sensitivity may be particularly salient among Generation Z consumers, as digitally mediated peer evaluation contexts heighten concerns about social recognition and reputational threat. This generational characteristic provides a contextual foundation for examining how impulse-buying propensity relates to face sensitivity in sports consumption settings.

Impulse buying is commonly defined as an unplanned, spontaneous purchase decision made at the point of sale (Kollat and Willet, 1969). While early research acknowledged that such decisions may involve situational evaluation, impulse buying has primarily been conceptualized as a consumption behavior driven by immediate emotional and environmental stimuli (Rook, 1987; Verplanken and Herabadi, 2001). In contemporary retail and digital contexts, marketers strategically design environmental cues to trigger these spontaneous responses, contributing to the rapid growth of impulse-buying research over the past decade (Muhammad et al., 2024; Redine et al., 2022).

Importantly, the defining characteristics of Generation Z—such as high digital immersion, continuous social media engagement, and heightened sensitivity to peer feedback—are closely aligned with the key variables examined in this study (Djafarova and Bowes, 2021). Digital platforms amplify opportunities for social comparison, public visibility, and real-time validation, thereby intensifying concerns related to social-face sensitivity (Vaid et al., 2024). Simultaneously, identity presentation and symbolic self-expression are frequently enacted through consumption choices, particularly in highly visible product categories such as sports apparel and merchandise (Jin and You, 2018). These contextual features make Generation Z an especially relevant population for examining the relational associations among impulse buying, social-face sensitivity, and conspicuous consumption tendencies (Theocharis and Tsekouropoulos, 2025).

Impulse buying refers to consumption arising spontaneously from desires and emotions without prior planning, and this phenomenon is becoming increasingly prominent in the consumption behavior of sports consumers (Chen et al., 2025). Recent studies have linked sports consumption to emotional stimulation, social identification, and instant gratification on digital platforms (Chen et al., 2025; Liu et al., 2025). In particular, the atmosphere and live experiences of sporting events have been shown to intensify participants’ purchase impulses, leading to the consumption of sports-related products such as merchandise, apparel, and equipment (Cho et al., 2025; Gârdan et al., 2020; Hwang et al., 2024). In this context, impulse buying among sports consumers can be interpreted not merely as irrational consumption, but as a consumption pattern that has been discussed as being related to sporting experience and brand engagement (Kwon and Armstrong, 2006).

Impulse buying behavior of sports consumers is influenced not only by individual factors but also by social contexts, with face sensitivity being a particularly important factor (Sun et al., 2021). Face sensitivity refers to the tendency to be acutely aware of others’ evaluations and perceptions (Jin et al., 2016) and is associated with stronger motivation to maintain a positive image within a group or to display social status (Ivanic, 2015). It has been demonstrated that participants who exhibit higher levels of impulse buying tend to demonstrate stronger average levels of face sensitivity in comparison to those who exhibit lower levels of such tendencies (Kim, 2021). This phenomenon may be conceptually linked to the close association between spontaneous consumption and social image management. Sports settings are characterized by collective identification and active social interaction, where participants place importance on face and are likely to impulsively purchase visible products such as merchandise, uniforms, and equipment (Cho et al., 2025). Relatedly, fan-based consumption can reflect group-bound orientations; for example, research on sport team fans’ ethnocentric consumption tendencies highlights that group-related identity frames can shape preferences and consumption choices within sport markets (Yurtsızoğlu and Gül, 2022). Sports consumption research further suggests that identity-based mechanisms operate through club-related meanings and fan group affiliation: perceptions of a club’s image and formal/informal fan-group membership have been shown to differentiate sports consumption behaviors among fans (Kural and Karataş, 2023). Such impulse buying may co-occur with psychological motives related to preserving and enhancing social face (Rifkin et al., 2023).

In sports consumption contexts, products such as uniforms, merchandise, and high-priced equipment often carry symbolic and identity-expressive value beyond functional utility (Papadimitriou and Apostolopoulou, 2015). Such visibility-oriented characteristics render sports goods particularly susceptible to conspicuous consumption motives, defined as the deliberate purchase of products to signal status and symbolic identity (O'cass and McEwen, 2004). Given that impulse buying propensity reflects heightened responsiveness to emotional and situational cues (Rook, 1987; Verplanken and Herabadi, 2001), individuals with higher impulsive tendencies may be more inclined to engage in visibility-based consumption patterns when exposed to socially salient sports environments (Kathuria and Bakshi, 2025). Accordingly, impulse-buying propensity appears theoretically associated with variations in individuality-seeking, trend orientation, brand orientation, and high-price preference within sports-related purchasing contexts (Chen et al., 2025).

The present study seeks to extend existing impulse-buying and social-face theories by examining how impulse-buying propensity differentiates patterns of social-face sensitivity and conspicuous consumption within sports product consumption contexts. Although prior research has investigated these constructs independently, their multidimensional interrelations have rarely been analyzed within a unified analytical framework, particularly in visibility-oriented domains such as sports consumption.

This gap is theoretically significant because sports products are highly symbolic and publicly displayed goods, rendering them especially susceptible to identity signaling and social evaluation processes (O'cass and McEwen, 2004; Papadimitriou and Apostolopoulou, 2015). The relevance of this inquiry is further amplified among Generation Z consumers, whose consumption practices are embedded in digitally mediated environments characterized by continuous peer comparison, self-presentation, and social validation (Vogel et al., 2014; Nesi and Prinstein, 2015). In such contexts, impulse-buying propensity may be systematically associated with heightened evaluative sensitivity and visibility-oriented consumption motives.

Moreover, as social-face sensitivity is culturally reinforced within collectivist societies, South Korea provides a theoretically grounded setting in which digitalization, social evaluation, and identity-oriented consumption converge. Accordingly, this study investigates whether multidimensional differences in social-face sensitivity and conspicuous consumption tendencies emerge according to levels of impulse-buying propensity among South Korean Generation Z sports consumers. By adopting a group-comparison design, the study contributes to a more integrated and culturally situated understanding of relational consumption dynamics in contemporary sports markets.

## 2 Literature review

### 2.1 Impulse-buying theory

Impulse buying refers to unplanned consumption behavior that occurs spontaneously without rational consideration and may often lead to post-purchase regret (Flight et al., 2012; Xiao and Nicholson, 2013). Verplanken and Herabadi (2001) identified “lack of planning” and “emotional response” as two fundamental elements of impulse buying, conceptualizing them in terms of cognitive aspects (lack of planning) and emotional aspects (emotional reactions before, during, and after purchase). Impulsive behavior has also been explained through individual personality traits, with impulsivity defined as the tendency to act immediately without sufficient consideration of the consequences (Sharma et al., 2014). Previous research has shown that higher levels of impulsivity are closely associated with impulse buying experiences (Dhandra, 2020). Therefore, unlike planned purchasing, impulse buying can be understood as a consumption behavior triggered by the complex interplay of internal emotions and external stimuli without prior deliberation (Amos et al., 2014). Importantly, the multidimensional structure of impulse buying—particularly the elements of emotional responsiveness and lack of planning—suggests meaningful individual differences in susceptibility to affective and social stimuli. Individuals characterized by stronger emotional reactivity and lower deliberative control may be more sensitive to social evaluation and symbolic consumption cues (Chen S. et al., 2022; Yu, 2022). Accordingly, impulse-buying propensity can function as a theoretically relevant grouping variable for examining whether variations in social-face sensitivity and conspicuous consumption tendencies emerge between individuals with higher versus lower impulsive orientations (Xu et al., 2022).

Several theoretical frameworks have been applied to explain impulse buying. For instance, Verplanken and Sato (2011) provided an integrative understanding of impulse buying from the perspective of psychological self-regulation, suggesting that consumers may use impulsive purchases to seek pleasure or to compensate for deficits in self-identity. According to dual-process theory, impulse buying emerges from an imbalance between the impulsive system, which is automatic and emotional, and the reflective system, which is deliberative and inhibitory (Strack and Deutsch, 2004; Tifferet and Herstein, 2012). Similarly, impulsivity theory and self-control theory emphasize that low self-control and high impulsivity are critical determinants of impulse buying behavior (Baumeister, 2002; Vohs and Faber, 2007).

In the sports consumption context, the excitement of live sporting events and collective identification operate as salient external stimuli that heighten emotional arousal and reduce deliberative processing, thereby increasing the likelihood of impulse purchasing (Hwang et al., 2024). These situational characteristics are particularly relevant to the core sub-dimensions of impulse buying, namely emotional responsiveness and lack of planning. In highly stimulating sports environments, individuals who are more emotionally reactive and less inclined toward cognitive deliberation may respond differently to social and symbolic consumption cues (Chen et al., 2025; Verplanken and Herabadi, 2001).

Accordingly, distinguishing between individuals with higher versus lower impulse-buying propensity provides a theoretically grounded basis for intergroup comparison (Aquino and Lins, 2023; Choi and Bum, 2020; Grisetto et al., 2021; Guo et al., 2024). Examining whether variations in social-face sensitivity and conspicuous consumption tendencies emerge across these groups enables a more nuanced understanding of how the sub-dimensions of impulsive purchasing behavior manifest within sports product consumption.

### 2.2 Social face theory

The theory of social face originates from Goffman’s (1955) conceptualization of face-work, which describes individuals’ efforts to maintain a positive social image and avoid embarrassment or loss of prestige in interpersonal interactions. Subsequent developments, including Brown and Levinson’s (1987) distinction between positive and negative face, expanded this perspective into a broader framework explaining communication behaviors shaped by social evaluation and normative expectations.

Within East Asian contexts, the concept of face (chemyon) has been refined into the construct of social-face sensitivity, defined as a psychological tendency to react sensitively to others’ evaluations and to regulate behavior in order to prevent face loss (Choi and Lee, 2002; Kim, 2021). Empirical research in Korean consumer studies has demonstrated that social-face sensitivity is positively associated with conspicuous consumption, preference for high-end brands, trend-seeking behavior, and reference group consciousness (Jin and You, 2018; Kim, 2021).

Importantly, these dynamics are particularly salient in sports consumption contexts, where products such as uniforms, merchandise, and equipment are highly visible and symbolically expressive (Noh and Ahn, 2025). In such environments, consumption decisions are often publicly displayed and socially evaluated, making face-related concerns more pronounced (Law et al., 2021; Zhigang et al., 2022). This relevance is amplified among Generation Z, whose consumption practices are embedded in digitally mediated environments characterized by continuous peer comparison, social validation, and identity presentation (Le and Ngoc, 2024; Shin et al., 2021). Accordingly, social-face sensitivity provides a theoretically grounded lens for examining how impulse-buying propensity may be associated with conspicuous consumption tendencies within contemporary sports markets.

A synthesis of preceding studies reveals that social face theory originates from the symbolic interactionist tradition, emphasizing individuals’ efforts to preserve face in interactive situations (Goffman, 1955; Inglis and Thorpe, 2023; Johansson, 2007). Within Korean and East Asian contexts, this framework has been operationalized into a multidimensional construct of social-face sensitivity (Hur and Chun, 2023; Jin et al., 2016). Although this construct has been extensively examined in collectivist cultural settings, the broader concern for social evaluation and impression management extends beyond specific cultural boundaries, particularly within digitally mediated environments characterized by continuous peer visibility and social comparison (Le Blanc-Brillon et al., 2025). However, the degree to which face sensitivity influences consumption behavior may vary across cultural contexts (Khan et al., 2024).

Despite the growing literature, research comprehensively examining how differences in face sensitivity manifest according to impulse-buying tendencies among Generation Z sports consumers remains limited. Therefore, this study seeks to refine understanding of these relationships within the South Korean sports market, while acknowledging the cultural specificity of the empirical setting.

### 2.3 Conspicuous consumption theory

Conspicuous consumption originates from Veblen’s (1899) status-signaling framework, which conceptualizes consumption as a publicly visible act aimed at securing relative social distinction. In contemporary digital environments, this signaling function is amplified through social media platforms, where visibility, peer comparison, and real-time feedback intensify the performative dimension of consumption (Bainotti, 2024; Shamu et al., 2024). For Generation Z—whose consumption practices are deeply embedded in digitally networked spaces—conspicuous consumption extends beyond price display to include brand symbolism, trend alignment, and online visibility (Rodrigues et al., 2021; Shin et al., 2021).

Within sports markets, highly visible products such as team uniforms, limited-edition footwear, and premium equipment function as socially recognizable identity markers (Chae et al., 2020). Given that impulse-buying propensity reflects heightened responsiveness to emotional and situational cues (Rook, 1987; Verplanken and Herabadi, 2001), digitally amplified visibility may be associated with a higher the likelihood that spontaneous purchasing decisions are directed toward symbolically expressive sports goods. In this sense, impulse buying and conspicuous consumption may converge in sports contexts, where acquisition and public display operate within a socially mediated cycle of evaluation and recognition (Noh and Ahn, 2025; Opelík et al., 2025). This digitally intensified signaling dynamic is also evident in emerging e-sports consumption research (Gul and Yurtsizoglu, 2025).

### 2.4 Generation Z’s FOMO syndrome regarding sports products

FOMO (Fear of Missing Out) is defined as a pervasive anxiety arising from concerns about being excluded from rewarding experiences, and is theoretically grounded in Self-Determination Theory as a response to unmet needs for autonomy, competence, and relatedness (Przybylski et al., 2013). Within digitally mediated environments, FOMO has been consistently associated with heightened social media engagement, perceived scarcity, and urgency-based decision making (Gupta and Sharma, 2021; Tandon et al., 2021).

In consumer behavior contexts, FOMO has been linked to impulsive purchasing, as perceived social exclusion and time-limited opportunities may reduce deliberative processing and amplify emotional responsiveness (Nguyen and Van Nguyen, 2025; Groenestein et al., 2024). Conceptually, FOMO aligns with social-face sensitivity in that both constructs reflect heightened awareness of peer evaluation and social positioning (Dinh and Lee, 2025). In highly visible product categories such as sports merchandise—where uniforms, limited editions, and athlete collaborations function as identity markers—FOMO may intensify the convergence of impulse-buying tendencies and conspicuous consumption motives (Choi et al., 2025; Opelík et al., 2025).

Generation Z, as digital natives embedded in continuous peer comparison structures, appears particularly susceptible to FOMO-driven consumption dynamics (Deloitte, 2023). However, in the present study, FOMO is not modeled as an antecedent or moderator variable. Rather, it is conceptualized as a contextual psychological climate that may amplify socially evaluative concerns and visibility-oriented consumption tendencies within South Korean Generation Z sports markets. This clarification ensures aim to improve precision while maintaining theoretical relevance.

Overall, impulse-buying tendency can be understood as a dispositional sensitivity to emotional and situational stimuli, social-face sensitivity reflects heightened concern regarding social evaluation, and conspicuous consumption captures visibility-oriented identity expression. In digitally mediated sports consumption contexts, these constructs are theoretically interconnected: individuals with higher impulse-buying propensity may exhibit greater responsiveness to socially evaluative cues, which in turn may align with stronger visibility-oriented consumption motives. This integrative perspective provides the conceptual basis for examining multidimensional group differences among South Korean Generation Z sports consumers.

## 3 Research hypotheses

Impulse buying often stems from emotional gratification and the desire for social recognition. Consequently, individuals with a high propensity for impulse buying may exhibit greater sensitivity to socially evaluative cues and may be more inclined toward consumption patterns associated with social signaling (Nghia et al., 2022; Rodrigues et al., 2021). Recent studies reaffirm that impulsive buying tendencies are not merely behavioral expressions but rather relatively stable psychological dispositions reflecting heightened emotional responsiveness and lower deliberative control. These characteristics appear particularly salient in the online and mobile consumption contexts of Generation Z, who are digital natives (Zhang et al., 2022; Redine et al., 2022; Guo et al., 2024). This perspective extends the classical discussions of Rook (1987), who defined impulse buying as “unplanned immediate purchasing behaviour,” and Rook and Fisher (1995), who conceptualized buying impulsiveness as a dispositional trait, to digitally mediated consumption environments (Rook and Fisher, 1995; Xie et al., 2025).

Recent studies on social-face sensitivity and conspicuous consumption tendencies report that consumers with higher impulsive buying tendencies tend to demonstrate stronger concerns regarding others’ evaluations and social comparison processes. In particular, sub-dimensions such as public self-consciousness may be relevant because individuals with higher emotional reactivity are likely to exhibit heightened awareness of how they are perceived by others, while social self-shaming may be associated with increased sensitivity to potential face loss in socially visible consumption settings (Kim, 2021). Within Generation Z’s digitally connected environments, constant peer comparison and real-time feedback may be associated with intensified face-related concerns and visibility-oriented consumption patterns (Ngo et al., 2025; Noh and Ahn, 2025).

Similarly, regarding conspicuous consumption, individuals with higher impulse-buying propensity may be more responsive to symbolic and socially recognizable consumption cues. Sub-dimensions such as individuality pursuit can be conceptually linked to the expressive function of spontaneous consumption, while trend orientation may reflect heightened responsiveness to rapidly circulating social signals within digital platforms. Brand orientation and preference for high-priced items may likewise be associated with status-relevant consumption motives in contexts where visibility and peer evaluation are salient (Rodrigues et al., 2021; Kim, 2021; Guo et al., 2024).

Building upon these theoretical and empirical observations, this study anticipates that within the Generation Z sports consumer group, significant differences may emerge in social-face sensitivity and conspicuous consumption tendencies depending on levels of impulse-buying propensity (high or low). Accordingly, the following hypotheses are proposed.

Given the multidimensional operationalization of both social-face sensitivity and conspicuous consumption, directional hypotheses are specified at the sub-factor level to enhance analytical precision.

*Hypothesis 1*: Social-face sensitivity:

*H1a*: Generation Z sports consumers with higher impulse-buying propensity will exhibit higher levels of Public Self-Consciousness than those with lower impulse-buying propensity.

*H1b*: Generation Z sports consumers with higher impulse-buying propensity will exhibit higher levels of Social Self-Shaming Tendency than those with lower impulse-buying propensity.

*H1c*: Generation Z sports consumers with higher impulse-buying propensity will exhibit higher levels of Social Formality Consciousness than those with lower impulse-buying propensity.

*Hypothesis 2*: Conspicuous consumption:

*H2a*: Generation Z sports consumers with higher impulse-buying propensity will demonstrate higher levels of Individuality-Seeking Orientation than those with lower impulse-buying propensity.

*H2b*: Generation Z sports consumers with higher impulse-buying propensity will demonstrate higher levels of Trend-Seeking Orientation than those with lower impulse-buying propensity.

*H2c*: Generation Z sports consumers with higher impulse-buying propensity will demonstrate higher levels of Brand Orientation than those with lower impulse-buying propensity.

*H2d*: Generation Z sports consumers with higher impulse-buying propensity will demonstrate higher levels of High-Price Orientation than those with lower impulse-buying propensity.

## 4 Materials and methods

### 4.1 Data collection procedure

The study was conducted in accordance with the approval of the Institutional Review Board (IRB) of the Korea National Institute for Bioethics Policy (KoNIBP) (Approval No. P01-202506-01-025). All procedures complied with ethical standards for research involving human participants. A non-probability convenience sampling strategy was employed to recruit Korean adults aged 20 years or older who had purchased sports-related products within the past year. Although Generation Z is generally defined as individuals born between 1995 and the early 2010s, the present study focused exclusively on the adult segment of this cohort due to ethical and legal considerations associated with surveying minors. Accordingly, the findings should be interpreted as reflecting older Generation Z consumers rather than the entire generational population.

Data collection was conducted over a two-month period, from 16 June to 25 August 2025. Recruitment took place at seven sports centers, two golf-related facilities, and five universities. As recruitment was conducted primarily in physical sports and educational settings, the sample may disproportionately reflect facility-attending and physically active individuals. Accordingly, the findings should be interpreted as representative of active, facility-engaged Generation Z sports consumers rather than the broader population of all Generation Z individuals, including purely online or non-participatory consumers. The study therefore adopts a context-specific rather than population-representative analytical perspective. Posters were displayed at the main entrances of each site, providing a brief description of the study purpose, eligibility criteria, participation procedures, and a QR code linking to the online survey. Interested individuals accessed the survey via the QR code or web link, reviewed detailed study information, and provided informed consent electronically before participation. The questionnaire was self-administered and completed online. The survey was developed and administered using Google Forms. All responses were securely stored in the principal investigator’s password-protected account to ensure confidentiality and restricted data access.

Prior to data collection, a power analysis was conducted using G*Power 3.1.9.7 for MANOVA (global effects, two groups, eight dependent variables), assuming a medium effect size, a significance level of 0.05, and statistical power of 0.80. The analysis indicated a minimum required sample size of 238 participants. A total of 250 responses were collected, and after excluding 16 incomplete or inconsistent questionnaires, 234 cases were retained for final analysis. Although the final sample size (*n* = 234) fell slightly below the minimum threshold suggested by the *a priori* power analysis (*n* = 238), the discrepancy was minimal (four cases). Given the small difference and the statistically significant multivariate results observed, the achieved statistical power is unlikely to have been substantially compromised. Nevertheless, this marginal shortfall should be acknowledged as a minor methodological limitation when interpreting the findings.

### 4.2 Research tools

This study employed the following four tools to empirically measure impulse buying tendency and related variables among South Korea’s Gen Z sports consumers. Each scale was adapted and refined based on previous studies to suit the context of sports product consumption. A 7-point Likert scale (1 = Strongly disagree to 7 = Strongly agree) was applied to quantitatively assess respondents’ tendencies.

In adapting the Impulse-Buying Tendency Scale, Social-Face Sensitivity Scale, and Conspicuous Consumption Scale to the sports goods consumption context, minor wording modifications were made where necessary to ensure contextual relevance (e.g., specifying “sports merchandise,” “sports apparel,” or “sports equipment” in place of general product references). No structural changes were made to the original factor configurations of the respective scales. Prior to the main survey, the adapted items were reviewed by four experts in sports marketing and consumer behavior to confirm content validity. A pilot test with a small sample of Generation Z sports consumers was conducted to assess clarity and comprehensibility, and only minor linguistic refinements were implemented based on the feedback.

#### 4.2.1 Demographic information

To describe the basic characteristics of the participants, demographic information was collected using four items: gender, age group (Generation Z subgroup), primary purchase channel, and main sport activity. In addition, participants were asked whether they had prior experience of impulse buying in sports-related purchases. Although this item is not a demographic characteristic per se, it was included as a behavioral screening variable to support subgroup classification and supplementary analyses.

#### 4.2.2 Impulse-Buying Tendency Scale

The term ‘impulse-buying tendency’ refers to a consumer’s tendency to make purchases in a spontaneous manner in response to emotional stimuli, without prior planning or deliberation (Verplanken and Herabadi, 2001). The scale is rooted in the theory of Rook (1987), and prior studies by Rook and Fisher (1995) and Beatty and Ferrell (1998) have explored the effects of emotional and normative factors on impulse buying. In this study, ‘impulse-buying scale’ developed by Verplanken and Herabadi (2001) was utilized to measure this tendency. This scale comprises nine items in a single-factor structure that capture emotional responses, lack of self-control, and impulsive judgment experienced by consumers during the purchase moment. Additionally, Minor wording adjustments were made to align item expressions with sports product purchasing contexts, while preserving the original conceptual structure. The dimensionality of the scale was subsequently verified through exploratory factor analysis.

#### 4.2.3 Social-Face Sensitivity Scale

‘Social-face sensitivity’ is defined as a psychological tendency to react sensitively to others’ perceptions and social evaluations, thereby adjusting one’s own behavior (Hope and Heimberg, 1988). Choi et al. (1994) categorized this construct into three sub-factors: public self-consciousness (PSC), social self-shaming tendency (SSST), and social formality consciousness (SFC). This multidimensional structure has been applied in sports consumer research (Yu, 2018; Jung et al., 2022).

In the present study, the original instrument was contextually refined to align with sports product purchasing situations. The scale underwent a structured content validation process involving four experts in sports marketing and related academic fields. Through iterative review and consensus, wording adjustments were made to enhance contextual relevance while preserving the conceptual definitions of PSC, SSST, and SFC.

The dimensional structure of the adapted scale was subsequently examined through exploratory factor analysis (EFA), and the results supported the retention of the three-factor configuration.

#### 4.2.4 Conspicuous Consumption Scale

Conspicuous consumption tendency is defined as a consumer’s deliberate purchase of expensive or branded products to signal social status or identity (Veblen, 1899). This study employed a 19-item scale based on Park and Park (2011), incorporating applications validated within sports consumption contexts (Kim et al., 2020; You and Jeong, 2023).

To ensure contextual suitability for sports product purchasing, the scale was refined through a structured content validation process involving four experts in sports marketing and related academic disciplines. Item expressions were adjusted to reflect sports-specific consumption contexts while maintaining the theoretical structure of the four sub-dimensions: individuality-seeking orientation (ISO), trend-seeking orientation (TSO), brand orientation (BO), and high-price orientation (HPO).

The construct structure of the adapted scale was subsequently assessed through exploratory factor analysis (EFA), and the results supported the expected four-factor solution.

### 4.3 Statistical analysis method

The present study involved the statistical analysis of 234 valid responses using SPSS version 27.0, following the procedures outlined below. First, frequency analysis was conducted to identify the participants’ basic demographic characteristics. Secondly, exploratory factor analysis (EFA) and Cronbach’s *α* coefficients were calculated to verify the validity and reliability of the measurement tools. Third, descriptive statistical analysis was conducted to calculate the means and standard deviations of the validated key constructs. Fourth, Pearson’s correlation analysis was undertaken to examine the relationships among the key variables. Fifth, Prior to conducting MANOVA, the underlying statistical assumptions were systematically examined. Normality was assessed by evaluating skewness and kurtosis values for each dependent variable, all of which fell within the acceptable range (±2), indicating approximate normal distribution. Multicollinearity among dependent variables was examined through correlation coefficients, which were below the recommended threshold of 0.80, suggesting no serious multicollinearity concerns. Homogeneity of covariance matrices was tested using Box’s M test, and the assumption was satisfied. Additionally, Levene’s test confirmed the homogeneity of variance across groups for each dependent variable. Finally, one-way MANOVA was performed to test for differences in social-face sensitivity and conspicuous consumption tendency according to impulse buying tendency groups and impulse buying experience. The significance level for all statistical analyses was set at *p* < 0.05.

## 5 Results

### 5.1 Demographic characteristics according to classification by level of impulse-buying tendency

Respondents completed the Impulse-Buying Tendency Scale developed by Verplanken and Herabadi (2001). For the purpose of group-based comparison using MANOVA, participants were divided into high and low impulse-buying groups based on a mean-split procedure using the overall sample mean (M = 3.40). Respondents scoring above the sample mean were classified as the high impulse-buying group (*n* = 106), whereas those scoring below the mean were assigned to the low impulse-buying group (*n* = 128). Demographic characteristics of respondents according to their classification by level of impulse-buying tendency are presented in Table 1.

**Table 1:** Demographic characteristics according to classification by level of impulse-buying tendency.

Classification		Group 1 (*n* = 106)	Group 2 (*n* = 128)
Gender	Male	60	80
	Female	46	48
Impulse buying experience	Yes	59	36
	No	47	92
Main purchase channel	Offline stores	39	54
	Online stores	56	61
	Official brand websites	6	10
	Secondhand trading platforms	5	3
Primary sport activity	Golf	30	37
	Fitness	32	19
	Running	18	20
	Yoga	8	12
	Ball sports	3	19
	Others	15	21
Total		234	

Group 1, high impulse-buying tendency; Group 2, low impulse-buying tendency.

Although dichotomizing a continuous variable through a mean-split procedure has been criticized for potentially reducing statistical power and attenuating effect sizes (MacCallum et al., 2002), subsequent methodological discussions have suggested that such procedures do not inherently inflate Type I error rates and may be acceptable under specific analytical conditions (Iacobucci et al., 2015). In the present study, the mean-split approach was adopted to operationalize impulse-buying propensity within a group-comparison framework, consistent with the segment-based analytical tradition in consumer behavior research (Rook and Fisher, 1995; Dhandra, 2020).

This grouping strategy was used to facilitate a theoretically motivated comparison of social-face sensitivity and conspicuous consumption tendencies across distinct levels of impulse-buying propensity, aligning with the study’s focus on dispositional segmentation within sports markets. Nevertheless, it is acknowledged that dichotomization may result in some loss of information and statistical sensitivity. Accordingly, the findings should be interpreted within this methodological boundary, and future research is encouraged to examine impulse-buying propensity as a continuous variable using regression-based or structural modeling approach.

### 5.2 Validity and reliability of research tools

The key variables observed in this study comprised impulse buying tendency, social-face sensitivity, and conspicuous consumption tendency. Employing exploratory factor analysis (EFA) was undertaken to verify the validity of each observed variable, and the internal consistency was calculated using Cronbach’s *α* coefficient to examine the reliability of the research tools. The factor analysis was performed using principal component extraction and varimax rotation. Each item was labeled according to its sequence (e.g., “Impulse Buying Tendency 1,” “Impulse Buying Tendency 2”).

#### 5.2.1 The validity and reliability of the Impulse-Buying Tendency Scale

As illustrated in Table 2, the results of the factor analysis for the scale employed to measure consumers’ impulse buying tendency are presented. The original measurement tool comprised nine items related to impulse buying tendency. The factor analysis results yielded a single factor without excluding any items, and this factor was named “impulse-buying tendency.” The factor loadings of all nine items were 0.4 and above, and the eigenvalue of this factor was 6.391, which was much higher than 1, ensuring the validity of the factor classification. Furthermore, Cronbach’s *α* for this single factor was 0.949, exceeding the threshold of 0.6 and thereby demonstrating the measurement tool’s reliability.

**Table 2:** The validity and reliability of the Impulse-Buying Tendency Scale.

Items	Factor loading	α
	1	
Impulse-buying tendency 7	0.895	0.949
Impulse-buying tendency 3	0.881	
Impulse-buying tendency 5	0.865	
Impulse-buying tendency 9	0.855	
Impulse-buying tendency 8	0.853	
Impulse-buying tendency 4	0.828	
Impulse-buying tendency 6	0.819	
Impulse-buying tendency 1	0.809	
Impulse buying tendency 2	0.773	

Factor name	Impulse buying tendency
Eigenvalue	6.391
Individual proportion of variance	71.014
Cumulative proportion of variance	71.014

KMO measure = 0.936, Bartlett’s unit matrix test = 1805.569 (df = 36, *p* < 0.001).

#### 5.2.2 The validity and reliability of the Social-Face Sensitivity Scale

As illustrated in Table 3, the factor analysis of the tool employed to measure social-face sensitivity yielded three factors, with no items excluded. Factor 1, comprising items related to sensitivity to social evaluation, vulnerability in self-efficacy, and internal conflict over autonomy infringement, was labeled “social self-shaming tendency.” Factor 2, encompassing items concerning an individual’s excessive sensitivity to others’ views, evaluations, and reactions, and the tendency to feel anxiety, embarrassment, and tension over the possibility of their external image or behavior being viewed negatively, was labeled “public self-consciousness.” Factor 3, consisting of items pertaining to the psychological tendency to value manners, formality, and dignity of speech and behavior in interpersonal and social situations, and to regulate self-expression accordingly, was labeled “social formality consciousness.”

**Table 3:** The validity and reliability of the Social-Face Sensitivity Scale.

Items	Factor loading			α
	1	2	3	
Social-face sensitivity 8	0.852	0.276	0.077	0.919
Social-face sensitivity 9	0.833	0.160	0.073	
Social-face sensitivity 6	0.804	0.343	0.056	
Social-face sensitivity 7	0.776	0.344	0.030	
Social-face sensitivity 11	0.743	0.366	0.044	
Social-face sensitivity 10	0.730	0.231	0.094	
Social-face sensitivity 2	0.291	0.900	0.053	0.959
Social-face sensitivity 1	0.280	0.880	0.092	
Social-face sensitivity 4	0.318	0.863	0.040	
Social-face sensitivity 5	0.322	0.856	0.120	
Social-face sensitivity 3	0.406	0.828	0.023	
Social-face sensitivity 14	0.039	0.111	0.871	0.882
Social-face sensitivity 16	−0.012	−0.051	0.856	
Social-face sensitivity 13	0.036	0.079	0.813	
Social-face sensitivity 15	0.030	0.136	0.810	
Social-face sensitivity 12	0.208	−0.014	0.767	

Factor name	SSST	PSC	SFC
Eigenvalue	4.333	4.313	3.450
Individual proportion of variance	27.081	26.956	21.562
Cumulative proportion of variance	27.081	54.037	75.599

KMO measure = 0.887, Bartlett’s unit matrix test = 3199.616 (df = 120, *p* < 0.001). SSST, Social self-shaming tendency; PSC, Public self-consciousness; SFC, Social formality consciousness.

The factor loadings of all items constituting each factor exceeded 0.4, and the eigenvalues of the three factors both exceeded 1, thereby confirming the factor classification’s validity. Additionally, the Cronbach’s α values calculated for each factor were 0.919 for social self-shaming tendency, 0.959 for public self-consciousness, and 0.882 for social formality consciousness, all exceeding the threshold of 0.6, thereby ensuring the reliability of the measurement tool.

#### 5.2.3 The validity and reliability of the Conspicuous Consumption Tendency Scale

As demonstrated in Table 4, the scale analysis employed to evaluate conspicuous consumption tendencies identified four factors: individuality-seeking orientation, high-price orientation, brand orientation, and trend-seeking orientation. Factor 1 was designated “Individuality-seeking orientation,” signifying the aspiration to express individuality through fashion selections. Factor 2 was designated “High price orientation,” denoting the aspiration to exhibit status through high-priced items. Factor 3 was named “Brand orientation,” reflecting a predilection for products from renowned brands. The final Factor 4 was labeled “Trend-seeking orientation,” denoting a predilection for the latest trends.

**Table 4:** The validity and reliability of the Conspicuous Consumption Tendency Scale.

Items	Factor loading				α
	1	2	3	4	
Conspicuous consumption tendency 3	0.899	0.057	0.189	0.170	0.926
Conspicuous consumption tendency 2	0.844	0.079	0.174	0.092	
Conspicuous consumption tendency 5	0.839	0.123	0.244	0.082	
Conspicuous consumption tendency 4	0.819	0.062	0.252	0.205	
Conspicuous consumption tendency 6	0.781	0.203	0.280	0.102	
Conspicuous consumption tendency 1	0.688	0.095	−0.016	0.262	
Conspicuous consumption tendency 18	0.129	0.900	0.110	0.152	0.930
Conspicuous consumption tendency 17	0.062	0.890	0.119	0.123	
Conspicuous consumption tendency 16	0.117	0.886	0.155	0.164	
Conspicuous consumption tendency 19	0.137	0.819	0.157	0.217	
Conspicuous consumption tendency 12	0.259	0.131	0.832	0.166	0.875
Conspicuous consumption tendency 15	0.174	0.226	0.788	0.215	
Conspicuous consumption tendency 14	0.174	0.224	0.778	0.274	
Conspicuous consumption tendency 13	0.278	0.032	0.728	0.129	
Conspicuous consumption tendency 10	0.082	0.122	0.093	0.812	0.810
Conspicuous consumption tendency 11	0.284	0.005	0.224	0.779	
Conspicuous consumption tendency 8	0.246	0.273	0.144	0.661	
Conspicuous consumption tendency 9	0.169	0.332	0.300	0.650	
Conspicuous consumption tendency 7	0.086	0.293	0.288	0.429	

Factor name	ISO	HPO	BO	TSO
Eigenvalue	4.420	3.540	3.044	2.752
Individual proportion of variance	23.263	18.634	16.021	14.483
Cumulative proportion of variance	23.263	41.898	57.918	72.401

KMO measure = 0.869, Bartlett’s unit matrix test = 3399.610 (df = 171, *p* < 0.001). ISO, Individuality-seeking orientation; HPO, High price orientation; BO, Brand orientation; TSO, Trend-seeking orientation.

The factor loadings of all items constituting each factor were 0.4 or above, while the eigenvalues of the two factors both exceeded 1, thereby confirming the validity of the factor classification. Additionally, the Cronbach’s α values calculated for each factor were 0.926 for individuality-seeking orientation, 0.930 for high price orientation, 0.875 for brand orientation, and 0.810 for trend-seeking orientation, all exceeding the threshold of 0.6, thereby ensuring the reliability of the measurement tool.

### 5.3 Descriptive statistics and correlation analysis for multicollinearity

As illustrated in Table 5, the following descriptive statistics and correlation analysis for multicollinearity are presented. Firstly, the descriptive statistical analysis demonstrated that the mean values of the key variables ranged from 5.15 (social-formality consciousness) to 3.91 (high price orientation). Furthermore, the absolute values of skewness and kurtosis were below 3 and 10, respectively, thus confirming that the normality assumption of the population was met. Consequently, the statistical validity for applying parametric statistical methods was established. Secondly, a correlation analysis was conducted among variables to examine multicollinearity.

**Table 5:** Descriptive statistics and correlation analysis for multicollinearity.

Classification		1	2	3	4	5	6	7	8
Impulse-buying tendency		1							
Social-face sensitivity	PSC	0.485^**^	1						
	SSST	0.396^**^	0.648^**^	1					
	SFC	0.023	0.151^*^	0.163^*^	1				
Conspicuous consumption tendency	ISO	0.303^**^	0.219^**^	0.261^**^	0.222^**^	1			
	TSO	0.385^**^	0.331^**^	0.420^**^	0.354^**^	0.464^**^	1		
	BO	0.432^**^	0.263^**^	0.401^**^	0.147^*^	0.511^**^	0.545^**^	1	
	HPO	0.331^**^	0.146^*^	0.279^**^	0.037	0.295^**^	0.487^**^	0.384^**^	1
Mean (M)		3.45	4.00	3.41	5.15	4.23	4.23	3.58	3.21
Standard deviation (SD)		1.53	1.59	1.17	1.21	1.40	1.21	1.41	1.60
Skewness		0.349	−0.204	0.278	−0.729	−0.255	−0.178	0.067	0.386
Kurtosis		−0.714	−0.762	−0.456	0.996	−0.412	−0.133	−0.517	−0.462

*p < 0.05, **p < 0.01. PSC, Public self-consciousness; SSST, Social self-shaming tendency; SFC, Social formality consciousness; ISO, Individuality-seeking orientation; TSO, Trend-seeking orientation; BO, Brand orientation; HPO, High price orientation.

### 5.4 Differences in social-face sensitivity and conspicuous consumption tendency according to impulse buying tendency (high and low groups)

In order to test Hypotheses 1 and 2, the assumptions underlying MANOVA were examined prior to analysis.

First, multivariate normality was assessed by examining skewness and kurtosis values for each dependent variable. All values fell within the acceptable range of ±2, indicating approximate normal distribution. Given the sample size (*N* = 234), minor deviations from normality were considered acceptable under the central limit theorem (see Table 5).

Second, multicollinearity diagnostics were conducted. Pearson correlation coefficients among dependent variables were below 0.80 (see Table 5).

Third, homogeneity of variance–covariance matrices was evaluated. Levene’s tests for equality of variances were non-significant for all dependent variables (*p* > 0.05), supporting the assumption of homogeneity of variances. Additionally, Box’s M test indicated that the covariance matrices were not significantly different across groups (Box’s M = 37.691, *F* = 1.303, *p* = 0.131), thereby satisfying the homogeneity of covariance assumption.

Having satisfied these assumptions, a one-way MANOVA was conducted, revealing a statistically significant multivariate effect (Wilks’ *Λ* = 0.712, *F* = 13.071, *p* < 0.001, *partial η^2^ = 0.288*). To further investigate the source of these differences, follow-up univariate ANOVAs were performed for each dependent variable. To control for the inflated Type I error rate associated with multiple testing, a Bonferroni adjustment was applied (*p* < 0.007). Significant group differences were observed in (a) public self-consciousness, (b) social self-shaming tendency, (c) individuality-seeking orientation, (d) trend-seeking orientation, (e) brand orientation, and (f) high-price orientation (see Table 6). Mean differences are presented in Table 7. Accordingly, H1a, H1b, H2a, H2b, H2c, and H2d were supported, whereas H1c was rejected.

**Table 6:** Differences in social-face sensitivity and conspicuous consumption tendency according to impulse buying tendency (High and Low Groups).

Scale	Factor	*df*	*F*	*p*	*η^2^*
Social-face sensitivity	Public self-consciousness	1	47.924	<0.001	0.171
	Social self-shaming tendency	1	35.750	<0.001	0.134
	Social formality consciousness	1	0.162	0.688	0.001
Conspicuous consumption tendency	Individuality-seeking orientation	1	10.195	0.002	0.042
	Trend-seeking orientation	1	32.860	<0.001	0.124
	Brand orientation	1	42.554	<0.001	0.155
	High price orientation	1	20.874	<0.001	0.083

*p* < 0.007.

**Table 7:** Mean scores for variables between groups based on impulse buying tendency.

	Social-face sensitivity			Conspicuous consumption tendency			
	1	2	3	4	5	6	7
Group 1	4.72	4.00	5.18	4.55	4.69	4.19	3.71
Group 2	3.40	2.92	5.12	3.97	3.84	3.08	2.79

Group 1 = High impulse-buying tendency; Group 2 = Low impulse-buying tendency; 1 = Public self-consciousness; 2 = Social self-shaming tendency; 3 = Social formality consciousness; 4 = Individuality-seeking orientation; 5 = Trend-seeking orientation; 6 = Brand orientation; 7 = High price orientation.

## 6 Discussion

The present findings should be interpreted within an integrative framework in which impulse-buying propensity functions as a dispositional driver, social-face sensitivity reflects evaluative reactivity, and conspicuous consumption represents identity-expressive outcomes in digitally mediated sports markets. This study examined whether differences in social-face sensitivity and conspicuous consumption tendencies emerged between South Korean Generation Z sports consumers with high versus low impulse-buying propensity. The MANOVA results revealed a statistically significant multivariate effect, indicating systematic differences between the two groups. Specifically, the high impulse-buying group demonstrated significantly higher levels of public self-consciousness and social self-shame tendency compared to the low impulse-buying group, whereas no significant difference was observed in social formality consciousness.

With respect to conspicuous consumption, the high impulse-buying group exhibited significantly stronger individuality-seeking orientation, trend-seeking orientation, brand orientation, and high-price orientation. These findings, within the South Korean cultural setting, suggest that impulse-buying propensity is associated with heightened sensitivity and stronger tendencies toward visibility-oriented and status-relevant consumption within sports product contexts. Importantly, social-face sensitivity is a culturally embedded construct rooted in collectivist traditions, where social evaluation, relational harmony, and external perception play central roles in identity formation (Markus and Kitayama, 1991). In such contexts, public self-consciousness and shame-related sensitivities may be more socially reinforced and normatively salient than in individualistic cultures, thereby potentially amplifying their association with visible consumption behaviors. However, these relational patterns should not be interpreted as broadly representative of all Generation Z consumers, as the findings are derived from a convenience sample of South Korean sports consumers embedded within a specific socio-cultural context.

### 6.1 Theoretical implications

Beyond the statistical group differences, the findings contribute to a more integrated understanding of how impulse-buying propensity, social-face sensitivity, and conspicuous consumption operate within digitally mediated sports consumption contexts. Rather than viewing these constructs as isolated tendencies, the results suggest a layered psychological structure.

Specifically, impulse-buying propensity may be understood as a dispositional orientation characterized by heightened emotional reactivity and reduced deliberative control in socially evaluative environments. Within this framework, public self-consciousness reflects heightened awareness of external evaluation, while social self-shaming tendency represents sensitivity to potential face loss. These evaluative sensitivities appear to amplify the likelihood that consumption may function as one possible avenue through which individuals manage perceived social positioning.

This interpretation aligns with prior evidence indicating that social comparison processes, anxiety, and low self-esteem are closely associated with impulsive purchasing in socially visible contexts (Rodrigues et al., 2021; Tran, 2022). Importantly, shame-related tendencies may be particularly relevant, as shame is linked to compensatory and affect-regulation behaviors in consumption settings. Individuals high in shame sensitivity may engage in impulsive purchasing not merely for hedonic reward, but as a means of managing perceived social inadequacy or evaluative threat (Martins et al., 2024; Kalal, 2023).

By contrast, the absence of significant differences in social formality consciousness suggests that norm-oriented face concerns—those grounded in hierarchy, etiquette, and restraint—operate through mechanisms distinct from reward-seeking or status-signaling consumption. Formality-based face sensitivity may be more closely aligned with behavioral regulation and conformity rather than with impulsive or visibility-oriented consumption (Jin et al., 2016; Shi et al., 2017).

Taken together, these findings suggest that not all dimensions of face sensitivity are equally implicated in impulsive consumption. Rather, evaluative-reactive components (public self-consciousness and social self-shaming) appear more strongly associated with impulsive and conspicuous consumption patterns than norm-conforming components (formality consciousness).

From a cultural-theoretical perspective, the present findings should be interpreted within the collectivist orientation of South Korean society, where the self is often defined relationally rather than independently (Markus and Kitayama, 1991). In collectivist settings, maintaining social face and managing external evaluation are integral components of social functioning. Consequently, face-related sensitivities may be more closely intertwined with consumption behaviors that carry public visibility and symbolic meaning. The stronger association between evaluative-reactive face dimensions and impulsive, conspicuous consumption observed in this study may therefore reflect culturally reinforced dynamics rather than universally applicable patterns.

Nevertheless, these interpretations should be approached cautiously. Given the cross-sectional and group-comparison design, the findings indicate statistical associations rather than directional psychological mechanisms. The proposed layered interpretation therefore remains theoretically grounded but empirically correlational.

### 6.2 Practical implications

Drawing upon the observed group-based differences, the present findings contribute to a more integrated understanding of how impulse-buying propensity is associated with socially evaluative and identity-expressive consumption orientations within digitally mediated sports markets.

First, higher levels of public self-consciousness (PSC) and social self-shaming tendency (SSST) among the high impulse-buying group suggest that impulsive purchasing may be relationally embedded in heightened sensitivity to social evaluation. Rather than viewing impulse buying solely as spontaneous consumption, the findings position it as a disposition statistically associated with socially visible identity management processes among South Korean Generation Z sports consumers. This interpretation aligns with prior research linking emotional reactivity and social comparison to impulsive consumption tendencies, but extends the literature by situating these dynamics within sports merchandise contexts characterized by high public visibility.

Second, the consistent elevation of all conspicuous consumption sub-dimensions (ISO, TSO, BO, HPO) in the high impulse-buying group reinforces the interpretation that impulsive purchasing propensity is associated with symbolic and identity-expressive consumption orientations. Within digitally networked environments, sports products operate not merely as functional goods but as socially communicative artifacts. The present findings therefore contribute to theoretical integration by demonstrating that impulsive disposition, social-face sensitivity, and conspicuous orientation coexist within a coherent relational framework in sports consumption contexts.

Third, the association between SSST and high impulse-buying propensity suggests that affective vulnerability may interact with socially visible consumption settings. In digitally mediated markets where peer comparison and real-time feedback are salient, emotionally charged consumption cues may be relationally linked to impulsive responses. Importantly, given the cross-sectional and group-comparison design, these interpretations reflect statistically observed associations rather than directional mechanisms.

Fourth, the non-significant difference in social formality consciousness (SFC) provides an important conceptual refinement. While PSC and SSST are evaluative-reactive constructs, SFC reflects norm adherence and hierarchical sensitivity. The absence of group differences suggests that not all dimensions of face operate similarly in impulsive consumption contexts, thereby clarifying the multidimensional structure of social-face sensitivity in sports markets.

Collectively, these findings advance prior literature by offering a synthesized framework in which impulse-buying propensity is positioned as a differentiating psychological orientation associated with socially evaluative sensitivity and identity-signaling consumption among South Korean Generation Z. However, the conclusions should be interpreted as culturally situated and relational in nature. Given that face sensitivity is socially constructed and differentially emphasized across cultural systems, the observed relational patterns may be particularly salient within collectivist environments such as South Korea. Accordingly, these findings should not be assumed to generalize to individualistic cultural contexts without further cross-cultural validation.

## 7 Conclusion

This study examined differences in social-face sensitivity and conspicuous consumption tendencies among South Korean Generation Z sports consumers according to their levels of impulse-buying propensity. The findings revealed a significant multivariate difference between high and low impulse-buying groups. Specifically, the high impulse-buying group demonstrated higher levels of public self-consciousness and social self-shame, whereas no significant difference was observed in social formality consciousness. In addition, all sub-dimensions of conspicuous consumption—individuality-seeking orientation, trend-seeking orientation, brand orientation, and high-price orientation—were significantly higher in the high impulse-buying group. These results suggest that impulse-buying propensity is systematically associated with socially evaluative sensitivity and visibility-oriented consumption patterns within sports product contexts.

The objective of the study was twofold: firstly, to contribute to academic discourse, and secondly, to provide practical implications for developing effective sports marketing strategies and approaches to impulse buying behavior.

In consideration of the presented findings, sports product marketers may consider developing context-sensitive strategies centered on digital platforms, emotional appeals, symbolic value, and identity expression. However, these implications should be interpreted within the correlational scope of the present cross-sectional design. These insights may be particularly relevant within collectivist cultural contexts similar to South Korea, and further empirical validation is required before extending them to different socio-cultural environments.

Overall, the study provides relational evidence that impulse-buying propensity differentiates socially oriented and symbolically expressive consumption patterns among South Korean Generation Z sports consumers within a collectivist cultural context. These findings should not be generalized beyond active, facility-attending South Korean Generation Z sports consumers, as the convenience sampling strategy and cultural specificity constrain the external validity of the results.

## 8 Limitations and future research

This study is subject to several limitations that should be acknowledged.

First, all constructs were measured using self-reported online questionnaires. Given the socially sensitive nature of social-face sensitivity and conspicuous consumption, responses may have been influenced by social desirability bias and impression management. Particularly within digitally visible Generation Z contexts, self-presentation tendencies may have shaped reported behaviors. Future research should incorporate behavioral data (e.g., transaction logs, purchase records), experimental manipulations, or observational approaches to triangulate findings beyond self-report measures.

Second, the study employed a non-probability convenience sampling strategy, recruiting participants primarily from sports facilities and university settings. This approach may have overrepresented physically active, institutionally affiliated, and facility-attending individuals while underrepresenting casual or purely online sports consumers. Accordingly, the external validity of the findings is limited, and the results should not be generalized to the broader Generation Z population, particularly those outside sports facility contexts or within different cultural environments. Future research should adopt probability-based sampling strategies and more diverse recruitment channels, including online panels and community-based sampling.

Third, the sample was restricted to South Korean Generation Z consumers embedded within a collectivist cultural context where social-face sensitivity is socially reinforced. As face-related constructs are culturally situated, the present findings should be interpreted as context-specific rather than universally generalizable. Cross-cultural comparative research is necessary to determine whether similar relational patterns emerge in individualistic or multicultural settings.

Fourth, the cross-sectional research design substantially limits causal inference. Although statistically significant group differences were identified, temporal precedence and directional pathways cannot be established. The observed associations should therefore be interpreted strictly as relational patterns rather than evidence of causal mechanisms. Longitudinal, experimental, or cross-lagged panel designs are recommended for future research.

Finally, although exploratory factor analysis (EFA) was conducted and internal consistency coefficients were high, confirmatory factor analysis (CFA) was not performed. Given that the scales employed in this study were adapted from previously validated instruments and applied within a group-comparison framework, construct validity was primarily assessed through EFA and reliability analysis. Nevertheless, the absence of CFA limits the ability to rigorously evaluate measurement model fit indices and to test competing factor structures. In addition, very high reliability coefficients may indicate potential item redundancy. Future research employing CFA or structural equation modeling would provide stronger evidence of construct validity and improve measurement precision. Taken together, these methodological and contextual constraints underscore the exploratory and context-bound nature of the present study.

## Data Availability

The datasets presented in this article are not readily available because the dataset contains personal survey responses and cannot be publicly shared to protect participants’ privacy and confidentiality. Requests to access the datasets should be directed to K-HJ, pure3241@khu.ac.kr.
